# The effect of a long-term treatment with cannabidiol-rich hemp extract oil on the adenosinergic system of the zucker diabetic fatty (ZDF) rat atrium

**DOI:** 10.3389/fphar.2022.1043275

**Published:** 2022-12-15

**Authors:** Gabor Viczjan, Anna Szilagyi, Barbara Takacs, Ignac Ovari, Reka Szekeres, Vera Tarjanyi, Tamas Erdei, Vanda Teleki, Judit Zsuga, Zoltan Szilvassy, Bela Juhasz, Balazs Varga, Rudolf Gesztelyi

**Affiliations:** ^1^ Department of Pharmacology and Pharmacotherapy, Faculty of Medicine, University of Debrecen, Debrecen, Hungary; ^2^ University of Debrecen, Doctoral School of Nutrition and Food Sciences, Debrecen, Hungary; ^3^ Department of Psychiatry, Faculty of Medicine, University of Debrecen, Debrecen, Hungary

**Keywords:** ZDF (zucker diabetic fatty), cannabidiol (CBD), heart, A1 adenosine receptor, receptorial responsiveness method

## Abstract

Cannabidiol (CBD), the most extensively studied non-intoxicating phytocannabinoid, has been attracting a lot of interest worldwide owing to its numerous beneficial effects. The aim of this study was to explore the effect that CBD exerts on the adenosinergic system of paced left atria isolated from obese type Zucker Diabetic Fatty (ZDF) rats, maintained on diabetogenic rat chow, received 60 mg/kg/day CBD or vehicle *via* gavage for 4 weeks. We found that N6-cyclopentyladenosine (CPA), a relatively stable and poorly transported A1 adenosine receptor agonist, elicited a significantly weaker response in the CBD-treated group than in the vehicle-treated one. In contrast, adenosine, a quickly metabolized and transported adenosine receptor agonist, evoked a significantly stronger response in the CBD-treated group than in the vehicle-treated counterpart (excepting its highest concentrations). These results can be explained only with the adenosine transport inhibitory property of CBD (and not with its adenosine receptor agonist activity). If all the effects of CBD are attributed to the interstitial adenosine accumulation caused by CBD in the myocardium, then a significantly increased adenosinergic activation can be assumed during the long-term oral CBD treatment, suggesting a considerably enhanced adenosinergic protection in the heart. Considering that our results may have been influenced by A1 adenosine receptor downregulation due to the chronic interstitial adenosine accumulation, an adenosinergic activation smaller than it seemed cannot be excluded, but it was above the CBD-naïve level in every case. Additionally, this is the first study offering functional evidence about the adenosine transport inhibitory action of CBD in the myocardium.

## 1 Introduction

Cannabidiol (CBD), the most extensively studied non-intoxicating constituent of the plant *Cannabis sativa*, differs from Δ^9^-tetrahydrocannabinol (THC), the best-known phytocannabinoid, only in the cleavage of a six-membered, oxygen-containing ring. CBD is widely labelled as a non-psychoactive compound (Bielawiec et al., 2020; Peng et al., 2022), while others, due to its anxiolytic, antipsychotic and antidepressant effects, consider CBD to be psychoactive but non-intoxicating (Kicman and Toczek, 2020).

The main source of CBD is hemp, which is a collective noun referring to one of the three major cultivar groups of *Cannabis sativa* (Ren et al., 2021). The list of well-evidenced and putative molecular targets of CBD in the human body contains more than fifty enzymes, ion channels, receptors and transporters^1^, interaction with which enables CBD to exert antiinflammatory, anticancer, neuroprotective, anticonvulsant, anxiolytic, antipsychotic, antidepressant, antidiabetic and even antiobesity effects (Bielawiec et al., 2020; Sunda and Arowolo, 2020; Kicman and Toczek, 2020; Bilbao and Spanagel, 2022; Peng et al., 2022). CBD is also reported to affect the adenosinergic system (Carrier et al., 2006; Liou et al., 2008; Pandolfo et al., 2011; Ibeas Bih et al., 2015; Gonca and Darici, 2015; Sunda and Arowolo, 2020). In terms of the myocardial function, the two most important targets of CBD may be the A_1_ adenosine receptor (evidenced in the heart (Gonca and Darici, 2015)) and the equilibrative and nitrobenzylthioinosine-sensitive nucleoside transporter (ENT1) (evidenced in neurons, macrophages, retinal and brain microglial cells (Carrier et al., 2006; Liou et al., 2008; Pandolfo et al., 2011)).

The A_1_ adenosine receptor, a member of the ubiquitous adenosine receptor family, mediates most of the protective and reparative effects of adenosine in the myocardium, including the well-measurable negative inotropic effect (Belardinelli et al., 1995; Fredholm et al., 2001; Szentmiklosi et al., 2011; Headrick et al., 2013; Lasley, 2018). In the supraventricular myocardium, adenosine is capable of reducing the contractile force even below the resting level (called direct negative inotropic effect) (Belardinelli et al., 1995; Fredholm et al., 2001).

ENT1, an important adenosine transporter in the cardiac muscle, can influence the distribution and levels of various nucleotides and exogenous nucleotide analogues (Deussen et al., 1999, 2006; Choi and Berdis, 2012; Boswell-Casteel and Hays, 2017). Beyond the molecular fit between substrate and carrier, the extent of this influence also depends on the rate and site of the metabolism of the given substrate. Adenosine is a quickly metabolizing substrate, net formation and elimination of which occur in the interstitium and cell interior, respectively (Deussen et al., 1999, 2006). Therefore, the adenosine transport is directed into the cells (e.g. endothelium and cardiomyocytes), furthermore its inhibition elevates the interstitial adenosine concentration (Deussen et al., 1999, 2006; Karsai et al., 2006). The significance of adenosine transport through ENT1 in the regulation of the interstitial adenosine level (and thereby in the adenosine-induced protective processes) is reflected by the repressed expression and activity of ENT1 during long-term ischemia (Choi and Berdis, 2012; Boswell-Casteel and Hays, 2017). The consequently elevated interstitial adenosine concentration enhances the adenosinergic signaling because of the cell-surface localization of the orthosteric binding site of adenosine receptors (Fredholm et al., 2001).

In summary, CBD may affect the myocardial adenosinergic signaling *via* at least two pathways: at the level of the A_1_ adenosine receptor (Gonca and Darici, 2015)^2^ and ENT1 (Carrier et al., 2006; Liou et al., 2008; Pandolfo et al., 2011). However, until now, the contribution of these pathways to the resultant effect of CBD remained unknown (Sunda and Arowolo, 2020; Ibeas Bih et al., 2015; Grubb et al., 2021). Therefore, the goal of the present study was to explore the main mechanism, by which CBD exerts its effects on the myocardial adenosinergic signaling. To address the problem of discriminating between adenosinergic activations caused by A_1_ adenosine receptor agonism and adenosine transport blockade, we applied a self-developed method enabling the correction of a concentration-effect (E/c) curve for the distortion caused by a surplus (and neglected) agonist concentration (Kiss et al., 2013; Zsuga et al., 2017; Erdei et al., 2018; Szabo et al., 2019a).

Based on the possible therapeutic benefits of CBD in both type 1 and type 2 diabetes mellitus that affect the heart as well (Rajesh et al., 2010; Bielawiec et al., 2020; Sunda and Arowolo, 2020; Peng et al., 2022), the essential experiments presented in this paper were carried out on isolated, paced atria of obese type Zucker Diabetic Fatty (ZDF) rats. The obese ZDF rat is a widely used animal model for the type 2 diabetes mellitus, a disease occurring with increasing frequency and being a major cause of blindness, kidney failure, heart attack, stroke, lower limb amputation and - in general - premature mortality worldwide^3^ (Henning, 2018). A “diseased” animal model may have greater translational potential as the results obtained this way can be more reliably applied to solving clinical problems. This is in accordance with the observation that some protective mechanisms, verified in a healthy state, do not prevail under certain pathological conditions (Juhasz et al., 2004).

## 2 Materials and methods

### 2.1 Materials

The following chemicals were used: adenosine and N^6^-cyclopentyladenosine (CPA), purchased from Sigma (St. Louis, MO, United States of America); a hemp extract oil rich in cannabidiol (CBD) and devoid of intoxicating psychoactive components, commercially available under the name “Vitality CBD Oral Drops/Spray 4800 mg Natural”, ordered directly from the manufacturer (Vitality CBD Ltd., Birmingham, UK)^4^; sunflower oil, commercially available under the name “Vénusz”, manufactured by Bunge CJSC (Martfű, Hungary).

CPA was dissolved in ethanol:water solution (1:4 v/v). Adenosine was dissolved in 36°C modified Krebs-Henseleit buffer (Krebs solution) containing (in mmol/L): NaCl: 118, KCl: 4.7, CaCl_2_: 2.5, NaH_2_PO_4_: 1, MgCl_2_: 1.2, NaHCO_3_: 24.9, glucose: 11.5, ascorbic acid: 0.1, dissolved in redistilled water. Both stock solutions were adjusted to a concentration of 10 mmol/L, and then were further diluted with Krebs solution. According to the manufacturer, the CBD-rich hemp extract in the product “vitality CBD” was dissolved in MCT (medium-chain triglyceride) oil. Before use, it was further diluted with sunflower oil in the required proportion.

### 2.2 Animal model and experimental groups

The animal use protocols were approved by the Committee of Animal Research, University of Debrecen, Hungary (5/2022/DEMÁB; 14 April 2022). Male, 10-week-old, lean type as well as obese type Zucker Diabetic Fatty (ZDF) rats were obtained from the AnimaLab Hungary Ltd (Vác, Hungary), the distributor of Charles River Laboratories International Inc (Wilmington, MA, United States of America). Until 6 months of age, the lean ZDF rats were maintained on conventional rat chow (S8106-S011 SM R/M-Z + H, purchased from the Toxi-Coop Ltd., Budapest, Hungary, the distributor of ssniff Spezialdiäten GmbH, Soest, Germany), while the obese ZDF rats were kept on a diabetogenic diet (Purina 5,008 rat chow, obtained from the AnimaLab Hungary Ltd., according to the recommendation of Charles River Laboratories International Inc.^5^).

The 6-month-old obese ZDF rats were randomized into two groups: the Obese ZDF group (*n* = 4) and the CBD-treated Obese ZDF group (*n* = 6), while the lean ZDF rats formed the Lean ZDF group (*n* = 8). In addition to continuing the previously introduced diets, animals in the Obese ZDF group received 0.2 ml of sunflower oil daily, *via* gavage, for 4 weeks, while animals in the CBD-treated Obese ZDF group received 60 mg/kg/day CBD, *via* gavage, in a 0.2 ml volume, for four weeks.

On the day before starting the administration of vehicle and CBD for the animals (*in vivo* treatments), the fasting blood glucose concentrations (in mmol/L) were (mean ± SEM): 5.9 ± 0.1, 19.1 ± 2.1 and 23.3 ± 1.4, while on the day after finishing the *in vivo* treatments, they were 5.6 ± 0.1, 19.3 ± 1.7 and 20.5 ± 1.7 in the Lean ZDF, Obese ZDF and CBD-treated Obese ZDF groups, respectively. Furthermore, at the beginning of the *in vivo* treatments, the body weights of animals (in g) were (mean ± SEM): 388.8 ± 7.6, 364.5 ± 25.8 and 368.1 ± 14.2, whereas at the end of the *in vivo* treatments, these values changed to 413.2 ± 8.2, 403.5 ± 28.9 and 398.4 ± 17.6 in the Lean ZDF, Obese ZDF and CBD-treated Obese ZDF groups, respectively. Thus, the obese type ZDF rats developed an advanced stage type 2 diabetes mellitus, in which condition their body weight was already slightly smaller than that of the lean controls (despite the “obese” and “lean” type names).

Before being sacrificed for this study, the animals in all the three groups underwent the following procedures to collect information regarding their *in vivo* functional status: Morris water maze test, oral glucose tolerance test, and then, under 100/10 mg/kg ketamine/xylazine anesthesia, electroretinography, electrocardiography and echocardiography. Data obtained from these investigations are planned to be used for further studies.

### 2.3 Preparations and protocols

The animals were guillotined and then left atria were quickly removed and mounted at 10 mN resting tension in 10 ml vertical organ chambers (Experimetria TSZ-04; Experimetria Kft, Budapest, Hungary) containing Krebs solution, oxygenated with 95% O_2_ and 5% CO_2_ (36°C; pH = 7.4). Atria were paced by platinum electrodes (3 Hz, 1 m, twice the threshold voltage) by means of a programmable stimulator (Experimetria ST-02; Experimetria Kft, Budapest, Hungary) and power amplifier (Experimetria PST-02; Experimetria Kft, Budapest, Hungary). The contractile force was characterized by the amplitude of the isometric twitches, which were measured by a transducer (Experimetria SD-01; Experimetria Kft, Budapest, Hungary) and strain gauge (Experimetria SG-01D; Experimetria Kft, Budapest, Hungary), and recorded by a polygraph (Medicor R-61 6CH Recorder; Medicor Művek, Budapest, Hungary).

Owing to the lack of any pretreatment with an agent affecting contractility, the direct negative inotropic effect evoked by the adenosine receptor agonists was measured. The direct negative inotropic response of an isolated, paced left atrium placed in a classic organ bath system (Weston et al., 2022) offers a simple and highly reliable opportunity of assessing the function of the myocardial A_1_ adenosine receptor.

### 2.4 E/c curve correction method used for the present study

If a neglected, surplus agonist concentration is present when an E/c curve is constructed with an agonist using the same signaling as the neglected agonist, then a distortion on this E/c curve (i.e. a virtual decrease in the effect) will develop. This E/c curve distortion is proportional to the magnitude of the surplus agonist concentration that enables the determination of this latter *via* curve fitting. This is the receptorial responsiveness method (RRM) (Gesztelyi et al., 2004; Grenczer et al., 2010a; 2010b) that can be performed with several regression setting options (Szabo et al., 2019b). RRM forms the base of another method designed to correct an E/c curve for the distortion caused by the surplus agonist concentration (Kiss et al., 2013; Zsuga et al., 2017; Erdei et al., 2018; Szabo et al., 2019a). An E/c curve corrected this way has two informative parts: the initial one (if the first agonist concentration, used for the E/c curve, elicits very little or no effect), and the final one (in the case of a saturated E/c curve, i.e. a curve with well-defined top plateau). The initial part shows the effect value evoked (solely or mainly) by the surplus agonist concentration (because of which the correction is needed). In turn, the final part of the corrected E/c curve indicates the real maximal effect value that has been falsified on the uncorrected E/c curve (Kiss et al., 2013; Zsuga et al., 2017; Erdei et al., 2018; Szabo et al., 2019a).

### 2.5 Empirical characterization of the E/c curves

All E/c curves were fitted to the Hill equation, a simple and reliable model of receptor function (Gesztelyi et al., 2012), that provided three empirical parameters to geometrically describe the E/c curves:

$$E=E_{\max}\cdot \frac{c^{n}}{c^{n}+EC_{50}^{n}}$$ (1)

where: E: the effect, herein defined as a percentage decrease in the initial contractile force of atria; c: the concentration of the agonist in the bathing medium (administered for the given E/c curve); E_max_: the maximal effect; EC_50_: the agonist concentration producing a half-maximal effect; n: the Hill coefficient (slope factor). For the regression, the default options of the software were used.^6^

### 2.6 Quantification of the distortion caused by CBD in the averaged CPA E/c curve

CBD, by inhibiting the inward myocardial adenosine transport (Carrier et al., 2006; Liou et al., 2008; Pandolfo et al., 2011), was assumed to accumulate surplus adenosine in the atrial interstitium and thereby to distort the E/c curves generated in the CBD-treated Obese ZDF group. The concentration of this surplus interstitial adenosine was quantified with RRM using the combination of two independent settings: individual *vs*. global fitting, and ordinary *vs*. robust fitting [a similar but more complex arrangement was applied recently: (Szabo et al., 2019b)]. During individual fitting, the averaged CPA E/c curve of the CBD-treated Obese ZDF group was fitted to Eq. 2, in which the three empirical parameters of the averaged CPA E/c curve of the Obese ZDF group (provided by fitting Eq. 1) had been substituted. When fitting globally, the averaged CPA E/c curves of both groups mentioned above were simultaneously fitted to Eq. 2, this time with variable empirical parameters.

$$E^{\prime }=100-\frac{100\cdot \left(100-E_{\max}\cdot \frac{{\left(c_{x}+c\right)}^{n}}{{\left(c_{x}+c\right)}^{n}+EC_{50}^{n}}\right)}{100-E_{\max}\cdot \frac{c_{x}^{n}}{c_{x}^{n}+EC_{50}^{n}}}$$ (2)

where: E’: the distorted effect (determined conventionally); E_max_, EC_50_, n: empirical parameters defining the CBD-naïve state of the “CPA–A_1_ adenosine receptor–atrial tissue” interaction [when fitting individually, these empirical parameters were fixed at constant values provided by Eq. (1), while during global fitting, these parameters were variable like c_x_ (see below)]; c: the concentration of CPA in the bathing medium (administered for the E/c curve); c_x_: the parameter indicating the CPA concentration that is equieffective with the surplus interstitial adenosine caused by CBD.

For every other regression setting, the default option was chosen.^7^

### 2.7 Correction of effect values of the averaged CPA and adenosine E/c curves distorted by CBD

The effect values of the averaged CPA and adenosine E/c curves of the CBD-treated Obese ZDF group were corrected using c_x_ obtained with individual and ordinary fitting [similarly to our previous E/c curve corrections (Kiss et al., 2013; Erdei et al., 2018)]. First, the effect value belonging to this c_x_ was determined using the Hill equation:

$$E_{x}=E_{\max}\cdot \frac{c_{x}^{n}}{c_{x}^{n}+EC_{50}^{n}}$$ (3)

where: E_x_: the effect evoked by the surplus interstitial adenosine produced by CBD; c_x_: the CPA concentration equieffective with the surplus interstitial adenosine produced by CBD; E_max_, EC_50_, n: the empirical parameters of the averaged CPA E/c curve of the Obese ZDF group (provided by fitting Eq. 1).

From this E_x_ and the distorted effect values of the averaged CPA and adenosine E/c curves of the CBD-treated Obese ZDF group, corrected effect values were computed using Eq. 4:

$$E=100-\frac{\left(100-E^{\prime }\right)\cdot \left(100-E_{x}\right)}{100}$$ (4)

where: E: the corrected effect value; E’: the distorted effect value; E_x_: the effect value calculated from c_x_ (by means of Eq. 3).

The corrected effect values reflect the combined effect of the surplus interstitial adenosine caused by CBD and the agonist (CPA or adenosine) administered during the generation of the given E/c curve. Non-etheless, the corrected effect values were plotted against solely the administered agonist concentrations since the exact value of the surplus interstitial adenosine produced by CBD could not be determined.

### 2.8 Data analysis

During the curve fitting, concentrations (c, EC_50_ and c_x_) were expressed as common logarithms.

Gaussian distribution of data and homogeneity of variances were tested with the Shapiro-Wilk test and Brown-Forsythe test, respectively. Since all datasets showed Gaussian distribution and homogenous variances, ordinary one-way ANOVA followed by Tukey post-testing was performed for comparison. The difference of means was considered significant at *p* < 0.05.

Curve fitting and statistical analysis were performed with GraphPad Prism 8.4.3 (686) for Windows (GraphPad Software Inc., La Jolla, CA, United States of America), while other calculations were made by means of Microsoft Excel 2016 (Microsoft Co., Redmond, WA, United States of America).

## 3 Results

### 3.1 Response to CPA and adenosine

**FIGURE 1:**
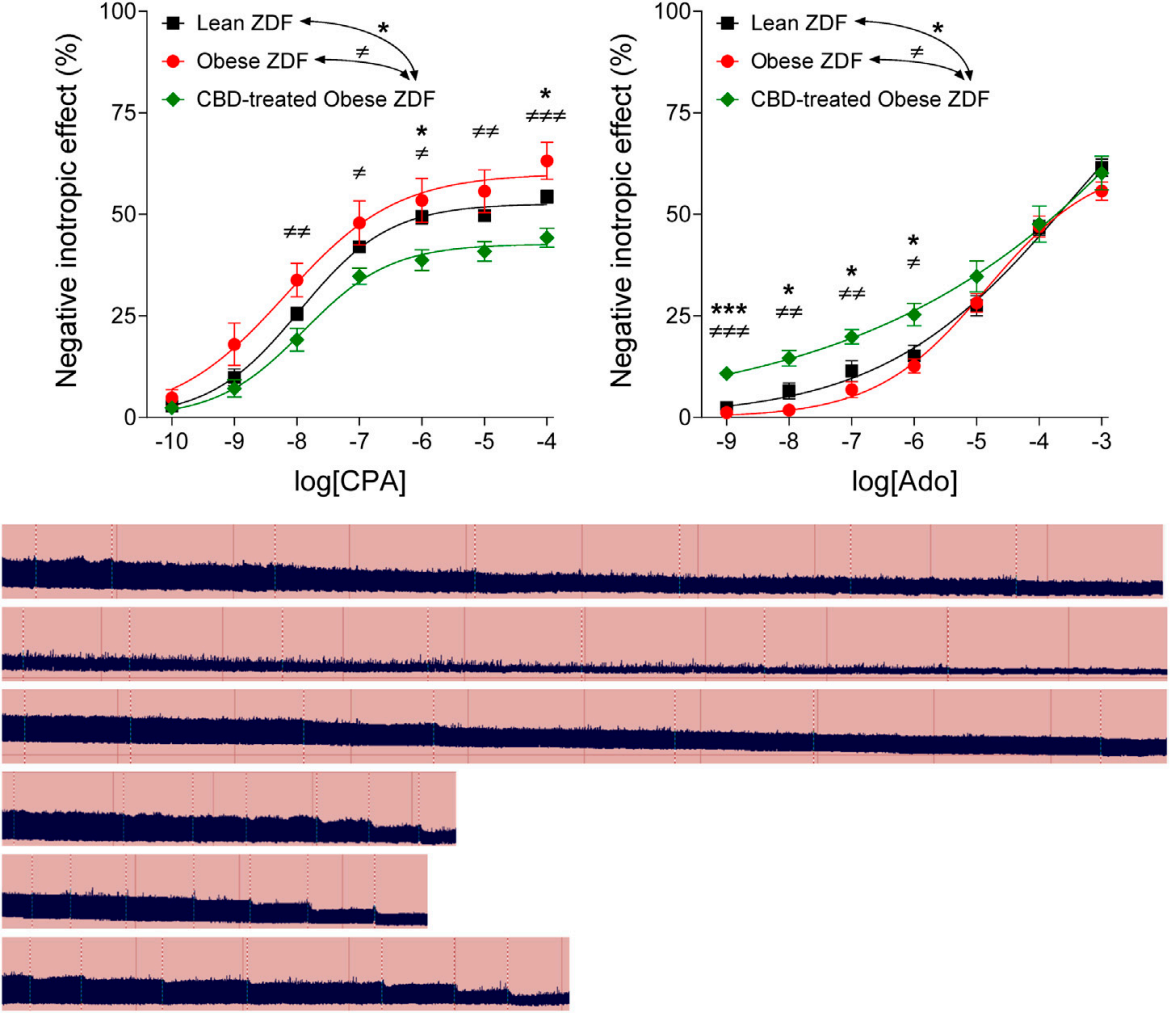
The direct negative inotropic effect of CPA and adenosine in isolated and paced atria of lean and obese types of Zucker Diabetic Fatty (ZDF) rats, without or with a previous *in vivo* cannabidiol (CBD) treatment (for the obese ZDF rats). On the top, two *x-y* graphs are presented, while below, six original records are shown (top three: response to CPA; bottom three: response to adenosine; in the order of the labels on the graphs). The *x*-axis denotes the common logarithm of the molar concentration of the given agonist (in the bathing medium), while the *y*-axis indicates the effect (as a percentage decrease in the initial contractile force). The symbols show the responses to the given agonist averaged within the groups (±SEM), and the curves illustrate the fitted Hill equation (Eq. 1). The vertical dotted lines on the original records denote the administration of the agonist doses. CPA: N^6^-cyclopentyladenosine; Ado: adenosine; *: comparison of the CBD-treated Obese ZDF group (n = 6) with the Lean ZDF group (*n* = 8); ≠: comparison of the CBD-treated Obese ZDF group with the Obese ZDF group (*n* = 4); the number of marks means the level of statistical significance (one mark: *p* < 0.05, two marks: *p* < 0.01, three marks: *p* < 0.001).

Both CPA and adenosine evoked a concentration-dependent decrease in the atrial contractile force in all groups. However, while CPA, a relatively stable, poorly transported, synthetic A_1_ adenosine receptor agonist, elicited the weakest response in the CBD-treated Obese ZDF group (Figure 1, left panel), adenosine, the quickly metabolized and transported physiological adenosine receptor agonist, evoked the strongest effect just in this group (Figure 1, right panel). In the Lean ZDF group, the behavior of atria towards CPA and adenosine was comparable to that observed in the Obese ZDF group (received vehicle treatment) (Figure 1).

### 3.2 Surplus interstitial adenosine produced by CBD

**FIGURE 2:**
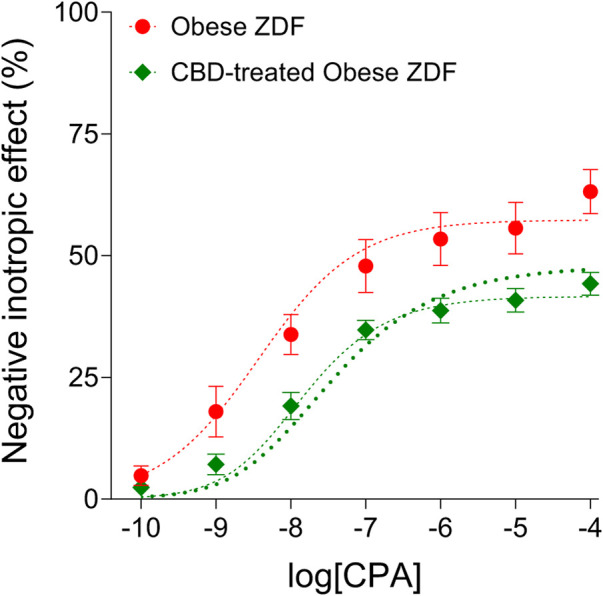
The direct negative inotropic effect of CPA in isolated and paced atria of obese type Zucker Diabetic Fatty (ZDF) rats, without or with a previous *in vivo* cannabidiol (CBD) treatment. The *x*-axis denotes the common logarithm of the molar CPA concentrations (in the bathing medium), and the *y*-axis indicates the effect (as a percentage decrease in the initial contractile force). The symbols show the responses to CPA averaged within the groups (±SEM). The thinner and the thicker dotted curves denote the model of the receptorial responsiveness method (RRM) (Eq. 2) fitted in a global plus robust manner and in an individual plus ordinary way, respectively. CPA: N^6^-cyclopentyladenosine.

The distortion seen on the averaged CPA E/c curve of the CBD-treated Obese ZDF group (shortly: the CBD-treated CPA E/c curve) as compared to the averaged CPA E/c curve of the Obese ZDF group (shortly: the intact CPA E/c curve) was evaluated with RRM, a method based on regression. The regression, performed with individual or global fitting in combination with ordinary or robust fitting (Figure 2), provided four c_x_ values as surrogates for the surplus interstitial adenosine caused by CBD, expressed as CPA concentrations being equieffective with it (Table 1). In line with our expectation, the four c_x_ values were within a narrow range, although one of them (resulted from global and ordinary fitting) was considerably smaller than the others. From the three c_x_ values being close to each other, we selected the one that was obtained with individual and ordinary fitting for further use. In addition, two further c_x_ values were yielded by the global fitting. These additional c_x_ values served as an internal control for the curve fitting, with zero as an expected value (because of the absence of CBD) (Table 1).

**TABLE 1:** The logc_x_ (and c_x_) values obtained with the receptorial responsiveness method (RRM) performed by combining two independent regression settings: individual *vs*. global, and ordinary *vs*. robust.

	Ordinary regression		Robust regression	
	To the intact curve	To the CBD curve	To the CBD curve	To the intact curve
Global fitting	−223 (≈0 nmol/L)	−8.712 (1.94 nmol/L)	−8.519 (3.02 nmol/L)	−223 (≈0 nmol/L)
Individual fitting	n.a	−8.568 (2.7 nmol/L)	−8.5 (3.2 nmol/L)	n.a

CBD, curve: the averaged CPA, concentration-effect (E/c) curve of the CBD-treated Obese ZDF, group; intact curve: the averaged CPA E/c curve of the Obese ZDF, group (treated with vehicle); CBD: cannabidiol; CPA: N^6^-cyclopentyladenosine; ZDF: zucker diabetic fatty; n. a. not applicable.

### 3.3 Corrected effects of the CPA and adenosine E/c curves generated in atria of CBD-treated rats

**FIGURE 3:**
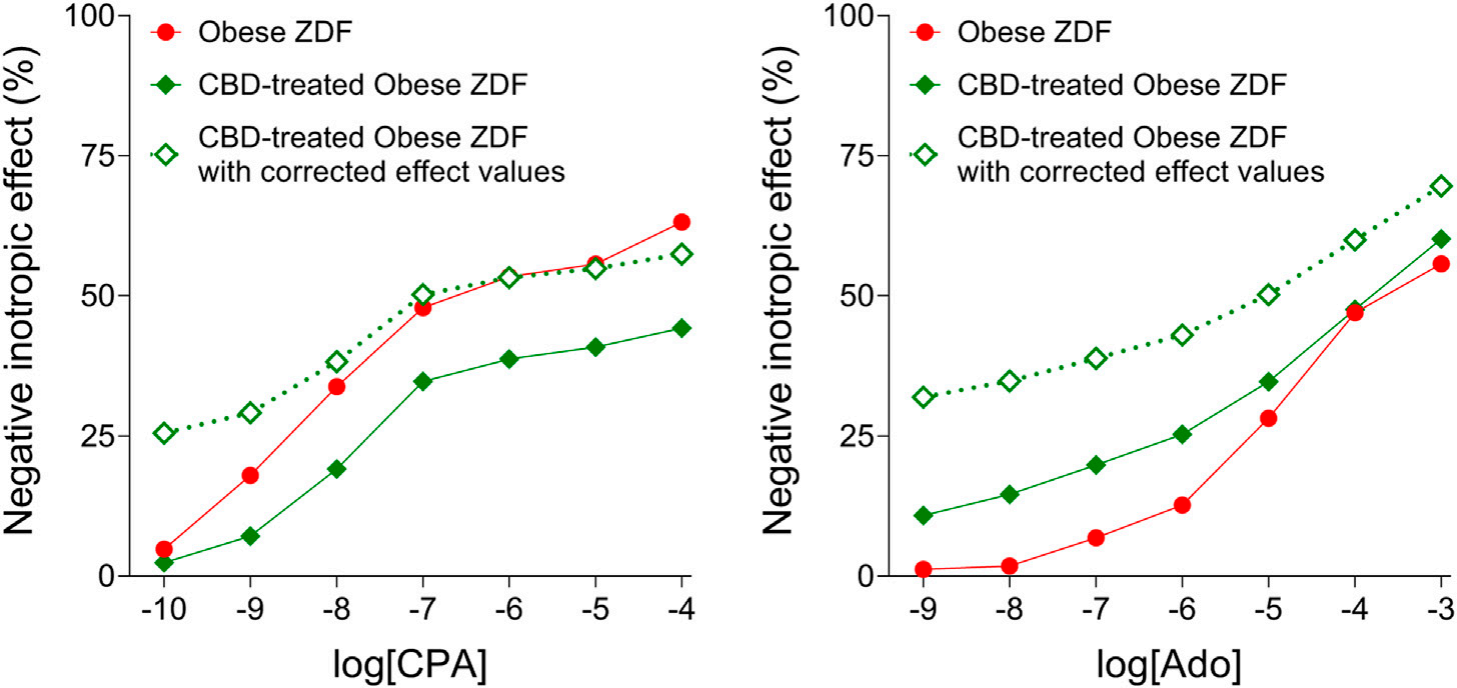
The corrected effect values of the averaged CPA and adenosine concentration-response (E/c) curves of the CBD-treated Obese ZDF group, furthermore the original (inherently correct) effect values of the averaged CPA and adenosine E/c curves of the Obese ZDF group (treated with vehicle). The *x*-axis shows the common logarithm of the molar concentration of the given agonist (in the bathing medium), while the *y*-axis indicates the effect (as a percentage decrease of the initial contractile force). The symbols represent the responses to the given agonist averaged within the groups. CPA: N^6^-cyclopentyladenosine; Ado: adenosine; CBD: cannabidiol; ZDF: Zucker Diabetic Fatty.

The corrected CBD-treated CPA E/c curve started from an about 25% effect value, indicating a considerably strong response that can be attributed to the surplus interstitial adenosine caused by CBD (Figure 3, left panel). Thus, CBD produced a “quarter-effective” adenosine concentration (EC_25_) in the atrial interstitium of the obese ZDF rats. The initial effect value of the corrected CBD-treated adenosine E/c curve was somewhat above 25% since the uncorrected initial effect value was unusually great, about 11% (Figure 3, right panel).

As expected, the final part of the corrected CBD-treated CPA E/c curve did not hold extra information in comparison with the final part of the intact CPA E/c curve. At low and medium concentrations, the corrected CBD-treated CPA E/c curve ran above the intact CPA E/c curve, whereas at high concentrations, they practically reached the same maximum (Figure 3, left panel). The result that the corrected and the intact CPA E/c curves shared approximately the same maximum meant that the data used were reliable.

In contrast, the corrected CBD-treated adenosine E/c curve considerably exceeded the intact adenosine E/c curve at all concentrations including the highest ones (Figure 3, right panel). Thus, the real maximal effect of the CBD-treated adenosine E/c curve was greater than the maximal effect of the intact adenosine E/c curve, indicating that the CBD treatment augmented the maximal response to adenosine.

Unfortunately, as the exact values of the interstitial adenosine concentration in the microenvironment of the myocardial A_1_ adenosine receptors remained unknown, the corrected effect values could only be plotted against the exogenous adenosine concentrations in the bathing medium (that could be computed) (Figure 3).

## 4 Discussion

To the best of our knowledge, this is the first study that has provided functional evidence about the adenosine transport inhibitory effect of CBD, a promising non-intoxicating phytocannabinoid, in the myocardium. The chronic oral administration of CBD to obese ZDF rats significantly increased the response of their isolated, paced left atria to adenosine, suggesting the presence of a long-term enhanced adenosinergic protection in the heart.

CBD has been being extensively investigated for a variety of indications owing to its antiinflammatory, antitumor, neuroprotective, anticonvulsant, anxiolytic, antipsychotic, antidepressant and antidiabetic properties (Bielawiec et al., 2020; Sunda and Arowolo, 2020; Kicman and Toczek, 2020; Bilbao and Spanagel, 2022; Peng et al., 2022). Moreover, CBD is commercially available as a dietary supplement. Thus, it is crucial to explore the consequences of the long-term use of CBD, with special regard to vital but vulnerable organs, such as the heart, and to fundamental protective mechanisms, like the adenosinergic system. Thus, in the present study, we focused on the effect that long-term oral CBD treatment exerted on the myocardial adenosinergic function, which was quantified by the robust direct negative inotropy characteristic of the atrium (Belardinelli et al., 1995; Fredholm et al., 2001). In the myocardium, most effects of adenosine, including the direct negative inotropy, are mediated by the A_1_ adenosine receptor (Belardinelli et al., 1995; Fredholm et al., 2001; Szentmiklosi et al., 2011; Headrick et al., 2013; Lasley, 2018).

Our CBD administration protocol aimed to model the human use of CBD, with special regard to the antidiabetic indication. In most human studies, the repetitive, oral administration of CBD was carried out with doses between about 1 and 50 mg/kg/day for 1–6 weeks(s) (Millar et al., 2019; Kicman and Toczek, 2020). Using the formula “rat dose = human dose ∙ 6.2” (Nair and Jacob, 2016), a rat dose range 6.2–310 mg/kg/day was obtained. In mice and rats, the most used repetitive administration regimens consisted of 10 mg/kg/day CBD, i. p. Or per os, for 1–6 weeks(s) (Wheal et al., 2017; Karimian Azari et al., 2020). Interestingly, i. p. And oral routes were found to result in similar concentrations in the rat (both in the plasma and brain) (Deiana et al., 2012). In diabetic rodent models, the duration of CBD treatment varied between 1 and 11 weeks(s), but the most frequent length was 4 weeks (El-Remessy et al., 2006; Weiss et al., 2006, 2008; Rajesh et al., 2010; Santiago et al., 2019). Taking all together, we decided to use 60 mg/kg/day CBD, per os, for 4 weeks, considering both the dose and duration to be sufficient to act in a well-detectable but safe (Dziwenka et al., 2020) manner.

In the present study, CPA, a relatively stable (Pavan and IJzerman, 1998) and thereby little transported, synthetic A_1_ adenosine receptor agonist (Fredholm et al., 2001, 2011), evoked a significantly smaller response in the atria isolated from the CBD-treated obese ZDF rats than in the atria of the vehicle-treated ones (Figure 1, left panel). In contrast, adenosine, the rapidly metabolized and transported physiological adenosine receptor agonist (Pavan and IJzerman, 1998; Fredholm et al., 2001, 2011), elicited a significantly greater response in the CBD-treated atria than in the vehicle-treated ones, but only at low and medium concentrations (Figure 1, right panel). According to the earlier experiences of our work team, this pattern is typical of the condition when the myocardial adenosine transport is blocked (Karsai et al., 2006; Kiss et al., 2013; Erdei et al., 2018; Viczjan et al., 2021). The reason for this is as follows: Upon sufficient oxygen supply, the myocardial adenosine transport is directed into the cells, so its inhibition increases the interstitial level of endogenous adenosine (Deussen et al., 1999, 2006; Karsai et al., 2006). This surplus interstitial adenosine, in part, uses up the response capacity of the adenosine receptors (prior to the generation of an E/c curve) (Gesztelyi et al., 2004). Hence, a poorly transported adenosine receptor agonist (like CPA) cannot evoke an effect as great as that seen with intact transport. Contrary to this, when a quickly metabolized and transported adenosine receptor agonist (like adenosine) is administered, two opposing effects prevail: the above-mentioned distorting effect that virtually reduces the response, and another effect that really augments the response, since the transport blockade protects also this metabolizable and transportable exogenous agonist from the intense intracellular elimination. As a result of these two opposing effects, enhanced responses to low and medium agonist concentrations, furthermore reduced or unchanged responses to high agonist concentrations develop. This was observed in our previous studies performed in guinea pig and rat atria, using nitrobenzylthioinosine derivatives for adenosine transport blockade and applying adenosine as a metabolizable and transportable exogenous agonist (Karsai et al., 2006; Kiss et al., 2013; Erdei et al., 2018; Viczjan et al., 2021). This is also the case in the present study as regards the CBD-treated atria: we have found weaker responses to all CPA concentrations (Figure 1, left panel), while stronger responses to low and medium adenosine concentrations and unchanged responses to high adenosine concentrations (in comparison with the vehicle-treated atria) (Figure 1, right panel).

Indeed, CBD was found to inhibit ENT1 (Carrier et al., 2006; Liou et al., 2008; Pandolfo et al., 2011). This inhibitory effect was reported to be relatively strong and concentration-dependent, to evolve from relatively low, 100 nM concentrations (Carrier et al., 2006; Ibeas Bih et al., 2015), and it was evidenced in neurons (Pandolfo et al., 2011), macrophages, retinal and brain microglial cells (Carrier et al., 2006; Liou et al., 2008), but not in the heart. Our present study is the first to provide functional evidence about the ENT1 inhibitory effect of CBD in the myocardium.

As adenosine activates all types of adenosine receptors, the possible involvement of the cardiac A_2A_ adenosine receptor should also be addressed. The A_2A_ adenosine receptor, primarily by stimulating the adenylyl cyclase, may increase the contractile force (Boknik et al., 2021). If CBD were able to attenuate the function of A_2A_ adenosine receptor (or its signaling), this mechanism could contribute to the stronger negative inotropic effect of adenosine after CBD treatment that was observed in the present study (Figure 1, right panel). However, to the best of our knowledge, no finding has yet been described to suggest this. Similarly, apart from suggestions (e.g. see Table 1 in Kicman and Toczek, 2020), no evidence about effects of CBD on activities of adenosine-handling enzymes has been found so far. Furthermore, if we deliberate the conceivable effects of CBD on the myocardial adenosinergic system other than nucleoside transport, none of them can explain our finding, i.e. CBD exerted opposite effects on the responses to adenosine and CPA (Figure 1).

To properly exhibit and evaluate the ENT1 inhibitory effect of CBD, some adjustment on the E/c curves representing the CBD-treated condition was needed. As mentioned above, if neglected, the presence of a surplus interstitial adenosine biases the conventionally evaluated and plotted E/c curves of adenosine receptor agonists, namely it causes a virtual (and not real) decrease in the response to these agonists (that can be spectacular or barely noticeable, depending on the circumstances). This distortion can lead to misinterpretations when comparing the affected E/c curves with undistorted (inherently correct) ones. To solve this problem, previously we elaborated a method, by which this E/c curve distortion can be corrected (Kiss et al., 2013; Erdei et al., 2018). With this method, it is possible to obtain E/c curve effect values that provide information on the magnitude of the cause of the distortion (looking at the initial part of the corrected E/c curve) and on the real receptorial responsiveness (looking at the final part of the corrected E/c curve).

The corrected CBD-treated CPA E/c curve started from an about 25% effect value, indicating that CBD accumulated circa the EC_25_ value of adenosine in the microenvironment of the myocardial A_1_ adenosine receptors (in terms of the direct negative inotropic effect) (Figure 3, left panel). Furthermore, the final part of the corrected CBD-treated adenosine E/c curve shows a considerably stronger maximal response than that of the vehicle-treated counterpart (Figure 3, right panel). These results imply that the long-term oral CBD treatment maintains a continuous, moderately elevated basal adenosinergic activity in the heart, furthermore it enhances the response to exogenous adenosine as well as to stimuli leading to adenosine release in the myocardial interstitium.

Nevertheless, it should be noted that our correction method ascribes all distortions manifested in a smaller response to an extra, unaccounted agonist concentration, which is here the surplus interstitial adenosine concentration accumulated by CBD (Figure 2). However, because of the chronic presence of a surplus adenosine, the issue of downregulation and/or desensitization of the A_1_ adenosine receptor should also be addressed. In a previous work, we found that the responsiveness of the myocardial A_1_ adenosine receptor did not decrease in the time window of our *ex vivo* experiments (Gesztelyi et al., 2004). Based on this, in our earlier studies dealing with the acute consequences of adenosine transport inhibition (Karsai et al., 2006; Kiss et al., 2013; Erdei et al., 2018; Viczjan et al., 2021), no decrease in the (real) responsiveness of the A_1_ adenosine receptor was considered. Consistent with this, the A_1_ adenosine receptor was reported to be desensitized extremely slowly, even in the presence of significant amounts of full agonists for several weeks (Willems et al., 2006; Mundell and Kelly, 2011). The 4-week CBD treatment applied in the present study, however, was long enough to consider the possibility of the desensitization of the A_1_ adenosine receptor. If there was some desensitization, the response to adenosine after the four-week CBD treatment may have been not as enhanced as it looks on the corrected adenosine E/c curve (Figure 3, right panel). The reason for this is that, upon desensitized receptors, the decrease in the response stems in part from the receptor desensitization, so the correction method overestimates the concentration of the surplus agonist that leads to an overcorrection of the distorted E/c curve. However, taking a look at the conventionally evaluated adenosine E/c curves (Figure 1, right panel), the long-term CBD treatment indisputably increased the response to adenosine at concentrations from 1 nmol/L to 10 μmol/L, an important range regarding cardioprotection (Headrick, 1996; Lasley and Mentzer, 1998) (So, what may be disputed here is only that the decreasing effect, which affected the even so enhanced response to adenosine, stemmed from solely the distortion mentioned above or from the coaction of the distortion and desensitization.) Thus, it can be concluded that the long-term oral CBD treatment significantly augmented the adenosinergic signaling of the heart even if the myocardial A_1_ adenosine receptors underwent some downregulation and/or desensitization.

The observation that the presence of CBD in the heart led to A_1_ adenosine receptor activation (Gonca and Darici, 2015) has raised the possibility that CBD might act as an A_1_ adenosine receptor agonist^8^. If so, CBD, persisting in the myocardium of the CBD-treated rats, had to act also as a surplus A_1_ adenosine receptor agonist. Based on this, CBD, as a surplus A_1_ adenosine receptor agonist, should have decreased the virtual response to both CPA and adenosine (indicated by a conventionally evaluated and plotted E/c curve). It should be noted that a surplus A_1_ adenosine receptor agonist (being present even before the E/c curve construction and being neglected) cannot exert any effect that would increase the (real or virtual) response to adenosine (or any other adenosine receptor agonist). However, in our present investigation, the *in vivo* CBD treatment decreased the response only to CPA, while it increased the response to adenosine (Figure 1). Thus, under our experimental conditions, CBD behaved as an adenosine transport blocker rather than an A_1_ adenosine receptor agonist. This result may offer strong evidence in the debate concerning whether the adenosine transport blocker or the adenosine receptor agonist property dominates the effect of CBD on the myocardial adenosinergic signaling.

As a limitation of this study, we should mention that, before being sacrificed, the animals used for this study underwent several *in vivo* investigations (for more details, see the Materials and Methods), which may have interfered with our measurements to some extent. Furthermore, the CBD-rich hemp extract oil, used for this study, contained other bioactive phytochemicals as well, effects of which may have contributed to our results. According to the manufacturer, this product contains cannabidivarin (0.038% w/w) in addition to CBD (16.802% w/w), and other components may also be present below the limit of quantification^9^. Based on results of others, cannabigerol, cannabichromene, Δ^8^-and Δ^9^-tetrahydrocannabinols, cannabinol, cannabicyclol (and their 3′-carboxy derivatives as “acids”), furthermore cannflavins, *β*-myrcene and *β*-caryophyllene may be the further components (Morales et al., 2017; Pellati et al., 2018; Ohtsuki et al., 2022). All have antioxidant property, most of them have been reported to exert antiinflammatory effects, and some have also been proposed to be neuroprotective (Morales et al., 2017; Scandiffio et al., 2020; Pannico et al., 2022). Importantly, the CBD-rich extracts appeared to be superior to the purified CBD products at the levels of both their beneficial and undesirable effects (Millar et al., 2020). Finally, the results of the present study rest on the assumption that the *in vivo* administered CBD was present in a sufficient amount in the atria to inhibit ENT1 during our *ex vivo* experiments. Our reason to assume this is the highly lipophilic nature of CBD. In our recent study, FSCPX, another highly lipophilic agent, was found to accumulate and persist in the lipid compartment (e.g. cell membranes) of the isolated atria, where it was able to exert its effects for a long time (Viczjan et al., 2021). Thus, it is reasonable to presume that CBD, administered *in vivo*, can persist in the atria long enough to elicit its effect *ex vivo* on the transmembranous ENT1.

## 5 Conclusion

In the present investigation carried out on atria isolated from obese ZDF rats, long-term oral CBD treatment has been found to significantly increase the negative inotropic response to adenosine but not CPA. This result, to the best of our knowledge, provides the first functional evidence of an adenosine transport inhibitory effect elicited by CBD in the myocardium, and suggests enhanced long-term adenosinergic protection in the heart. In association with this, we have demonstrated that, under our experimental conditions, the transport inhibitory action of CBD is dominant over its supposed A_1_ adenosine receptor agonist property in terms of the influence on the myocardial adenosinergic function. The merit of these results may be augmented by the fact that they were obtained from an animal model of type 2 diabetes mellitus, a health condition that puts a significant burden on the healthcare systems worldwide.

## Data Availability

The original contributions presented in the study are included in the article/Supplementary Material, further inquiries can be directed to the corresponding author.

## Footnotes

1
https://go.drugbank.com/drugs/DB09061 (accessed on 24 August 2022).

2
https://go.drugbank.com/drugs/DB09061#BE0000013 (accessed on 12 November 2022).

3
https://www.who.int/news-room/fact-sheets/detail/diabetes (accessed on 25 August 2022).

4
https://cdn.shopify.com/s/files/1/0085/2846/1905/files/Certificate_-_Vitality_CBD_Dual_applicator_4800mg_Natural_-_WO022343.pdf (accessed on 16 November 2022).

5
https://www.criver.com/products-services/find-model/zdf-rat-obese?region=3631 (accessed on 12 November 2022).

6
https://www.graphpad.com/guides/prism/8/curve-fitting/index.htm (accessed on 13 August 2022).

7
https://www.graphpad.com/guides/prism/8/curve-fitting/index.htm (accessed on 13 August 2022).

8
https://go.drugbank.com/drugs/DB09061#BE0000013 (accessed on 12 November 2022).

9
https://cdn.shopify.com/s/files/1/0085/2846/1905/files/Certificate_-_Vitality_CBD_Dual_applicator_4800mg_Natural_-_WO022343.pdf (accessed on 16 November 2022).
